# Efficient estimation of gadolinium‐based contrast agent concentration using transient‐state keyhole MR‐STAT

**DOI:** 10.1002/mp.70155

**Published:** 2025-11-19

**Authors:** Fei Xu, Edwin Versteeg, Hongyan Liu, Miha Fuderer, Oscar van den Heide, Wybe J. M. van der Kemp, Cornelis A. T. van den Berg, Alessandro Sbrizzi

**Affiliations:** ^1^ Computational Imaging Group for MR Diagnostics & Therapy Center for Image Sciences UMC Utrecht Utrecht the Netherlands; ^2^ Department of Radiology University Medical Center Utrecht Utrecht the Netherlands

**Keywords:** concentration estimation, contrast enhancement, gadolinium‐based contrast agent, MR‐STAT, quantitative imaging

## Abstract

**Background:**

Fast quantitative MRI (qMRI) techniques have emerged to quantify multiple tissue parameters from a single acquisition in clinically feasible scan times. To integrate fast qMRI techniques into a clinical MRI protocol, one needs to consider imaging after injection of a contrast agent (e.g., dynamic contrast‐enhanced [DCE] imaging). However, fully repeating the multi‐parametric scan after the injection requires lengthy scan times, which are impractical for tracking the contrast agent concentration.

**Purpose:**

This work aimed to develop and validate an accelerated multi‐parametric method capable of estimating the concentration of Gadolinium‐based contrast agents (GBCAs) in a more time‐efficient way.

**Methods:**

We designed an accelerated 2D Magnetic Resonance Spin TomogrAphy in Time‐domain (MR‐STAT) protocol and tested it on a 3T MRI scanner. This is achieved by combining transient‐state acquisitions with Cartesian keyhole‐based acceleration. Based on the reconstructed *T*_1_ values, GBCA concentrations were quantified in phantoms containing water, manganese chloride, and five different concentrations of gadobutrol (Gadovist), ranging from 0.05 to 0.9 mM. Linear regression analysis between the estimated GBCA concentration and the reference values was performed to assess the accuracy. The GBCA concentration quantification method was then applied for synthetic patient data and a hybrid human/phantom study to demonstrate the feasibility of this method.

**Results:**

The accelerated MR‐STAT protocol, with an undersampling factor of 4, provided accurate *T*_1_, *T*_2,_ and GBCA concentration measurements. Estimated GBCA concentrations were in a strong linear relationship with reference values, with a slope and intercept on simple linear regression analysis of 1.034 and 0.009, respectively.

**Conclusion:**

We present a multi‐parametric approach for quantitatively assessing the concentration of gadolinium in vitro and in a hybrid in vivo/in vitro controlled setup using the accelerated MR‐STAT protocol. The concentration of gadobutrol in the range of 0.05–0.9 mM could be measured in a clinically applicable scan time using the proposed method, but needs further in vivo validation.

## 1 INTRODUCTION

Quantitative magnetic resonance imaging (qMRI) aims primarily at revealing tissue properties such as relaxation times (*T*_1_, *T*_2_) and proton density (PD), which carry information about the local microstructural environment and can support disease characterization and monitor disease progression.^1^, ^2^, ^3^, ^4^, ^5^, ^6^, ^7^ Over the last decade, a number of fast qMRI techniques have emerged to quantify multiple tissue parameters from a single acquisition in clinically feasible scan times. Examples include MR fingerprinting,^8^ Quantitative Transient‐state Imaging (QTI),^9^ a three‐dimensional interleaved Look‐Locker sequence with *T*_2_ preparation pulse (3D‐QALAS),^10^ and Magnetic Resonance Spin TomogrAphy in Time‐domain (MR‐STAT).^11^ Additionally, quantitative tissue maps reconstructed with these techniques enable the synthesis of multiple contrast‐weighted images similar to those used in clinical routines within substantially reduced examination times.^12^, ^13^, ^14^

The advantage of qMRI's fast acquisition time makes it highly promising for application in dynamic contrast‐enhanced (DCE) clinical MRI protocols.^16^ However, its implementation requires consideration of even more efficient imaging after the injection of a contrast agent. Gadolinium‐based contrast agents (GBCAs) are widely employed to alter the relaxation properties of pathologic tissue and facilitate disease diagnosis.^17^, ^18^, ^19^ To monitor the kinetic behavior of GBCAs in vivo, it is necessary to correlate changes in concentration with the signal variations observed in MR images. Given the direct proportionality between the concentration of GBCAs and the change in 1/*T*_1_, a series of *T*_1_ measurements within tissue as the GBCAs distribute could be employed to monitor changes in GBCA concentration.^20^, ^21^ In the context of future exams where fast qMRI protocols are employed, the relaxometric information for tissue prior to the injection of contrast agent is already available. What is needed is a time‐efficient post‐injection relaxometric protocol that, when combined with the pre‐injection protocol, can deliver GBCA concentration values. This could be used to either synthesize post‐injection contrasts, such as *T*_1_‐weighted images, or to model the time‐dependent uptake of GBCA (DCE MRI).

We note that typically, only contrast information undergoes significant changes following GBCA injection. Hence, the post‐injection acquisition can be drastically undersampled, as the information needed for contrast changes is mainly encoded in the low spatial frequency components of the k‐space.^22^ We therefore seek a fast qMRI protocol which can efficiently deal with partial (low frequencies) encoding, such as a keyhole sampling strategy. The keyhole strategy presents challenges in MR‐STAT. Unlike conventional MRI, where all phase encoding lines are acquired with a steady‐state, a transient‐state flip angle train is used in MR‐STAT, meaning that each phase encoding line is acquired with different flip angles. This introduces additional complexity in matching the keyhole data to the ‘reference’ data, as they are not acquired with the same signal behaviour.^23^, ^24^ In this work, we propose a keyhole‐based MR‐STAT protocol by designing a sampling scheme that is fully sampled for the pre‐injection acquisition and only acquires the low‐frequency component for the post‐injection scan. The pre‐injection sampling partially mimics the post‐injection sampling by re‐ordering the phase encoding lines (typically linearly ordered) according to a so‐called “low‐high” strategy: first, the low frequencies are sampled (the same ones as for the post‐injection acquisition), followed by the remaining high frequencies (which are not sampled after the injection). This approach is essential to guarantee consistency of the transient‐state dynamics and phase encoding pattern throughout the acquisitions, ensuring that the low frequencies k‐space data for both pre‐ and post‐injection acquisition (i.e., the keyhole part) share the same encoding information. Consequently, changes observed in *T*_1_ maps primarily reflect the concentration of GBCA, minimizing the influence of potential confounding factors,^25^, ^26^, ^27^, ^28^ such as diffusion and magnetization transfer effect. We then optimize the transient‐state flip angle train with respect to this specific encoding scheme. We demonstrate the feasibility of estimating GBCA concentration with the optimized MR‐STAT protocol at 3T MRI by means of phantoms and hybrid human/phantom studies.

## 2 MATERIALS AND METHODS

### 2.1 Sequence design and optimizations

As significant GBCA‐induced changes occur mainly in the low frequencies of k‐space following the GBCA injection, we acquired the keyhole segment of the post‐injection MR‐STAT scan with its own transient‐state flip‐angle train. Consistency in flip angle and sampling pattern throughout the acquisitions in MR‐STAT is crucial. Therefore, we opted for a “low‐high” sampling approach also for the pre‐injection measurement and started with the same low‐frequency segment (time‐varying flip angle and key‐hole gradient encoding) of the post‐injection acquisition. The remaining high‐frequency components are encoded immediately after the low‐ones.

Next, we optimized the flip angle train for this low‐high sampling pattern using BLAKJac,^29^ which efficiently optimizes sequences in the context of a predetermined phase‐encoding pattern. BLAKJac evaluates the signal‐to‐noise ratio of the combined time‐varying RF train and gradient‐encoding scheme, enabling flip angle optimization for the given spatial encoding.

**FIGURE 1:**
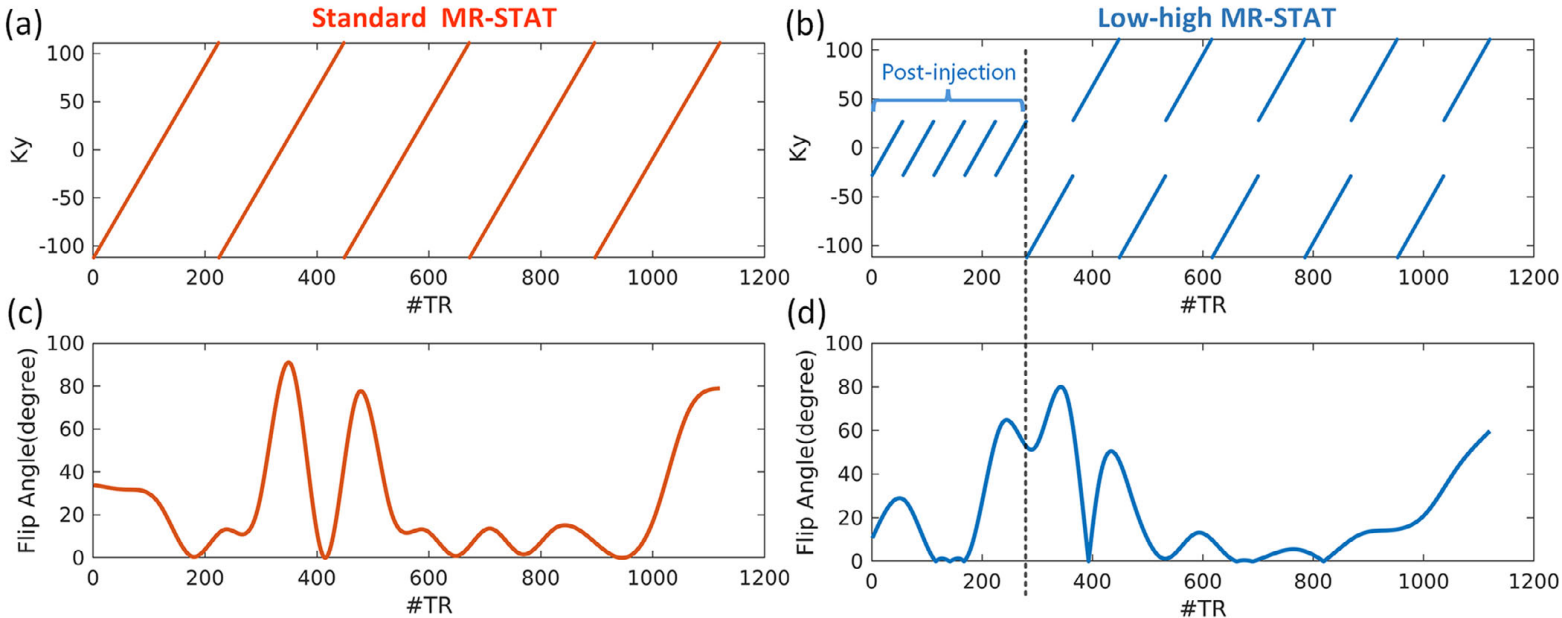
(a, b), sampling pattern used in the standard MR‐STAT and optimized low‐high MR‐STAT protocol; (c, d), the optimized flip angle train corresponding to two sampling patterns. After the GBCA injection, only the low‐frequency segment (first 280 repetitions) is applied in the optimized low‐high MR‐STAT protocol. GBCA, gadolinium‐based contrast agent; MR‐STAT, Magnetic Resonance Spin TomogrAphy in Time‐domain.

Once the optimal sequence for low‐high MR‐STAT was determined, it was applied to the pre‐injection acquisition, while only the low‐frequency components were utilized for the post‐injection acquisition. This ensures that two successive scans share identical encoding information of low‐frequency components of *T*_1_ and *T*_2_. See Figure 1b. The standard MR‐STAT sequence is shown in Figure 1a.

### 2.2 Imaging protocol

Quantitative MRI studies were performed using a 2D MR‐STAT sequence on a Philips 3T MR scanner. The MR‐STAT sequence is a spoiled gradient echo acquisition method with an inversion pulse, followed by time‐varying flip angles (Figure 1d), combined with a linearly ordered Cartesian sampling. The 2D imaging parameters (single slice) for all tests were: slice thickness: 3 mm; FOV: 224 × 224 mm^2^; in‐plane resolution: 1 × 1 mm^2^; TE = 4.5 ms; TR = 8.5 ms; acquisition time: 10 s for “pre‐injection” and 2.5 s for the accelerated “post‐injection”.

**FIGURE 2:**
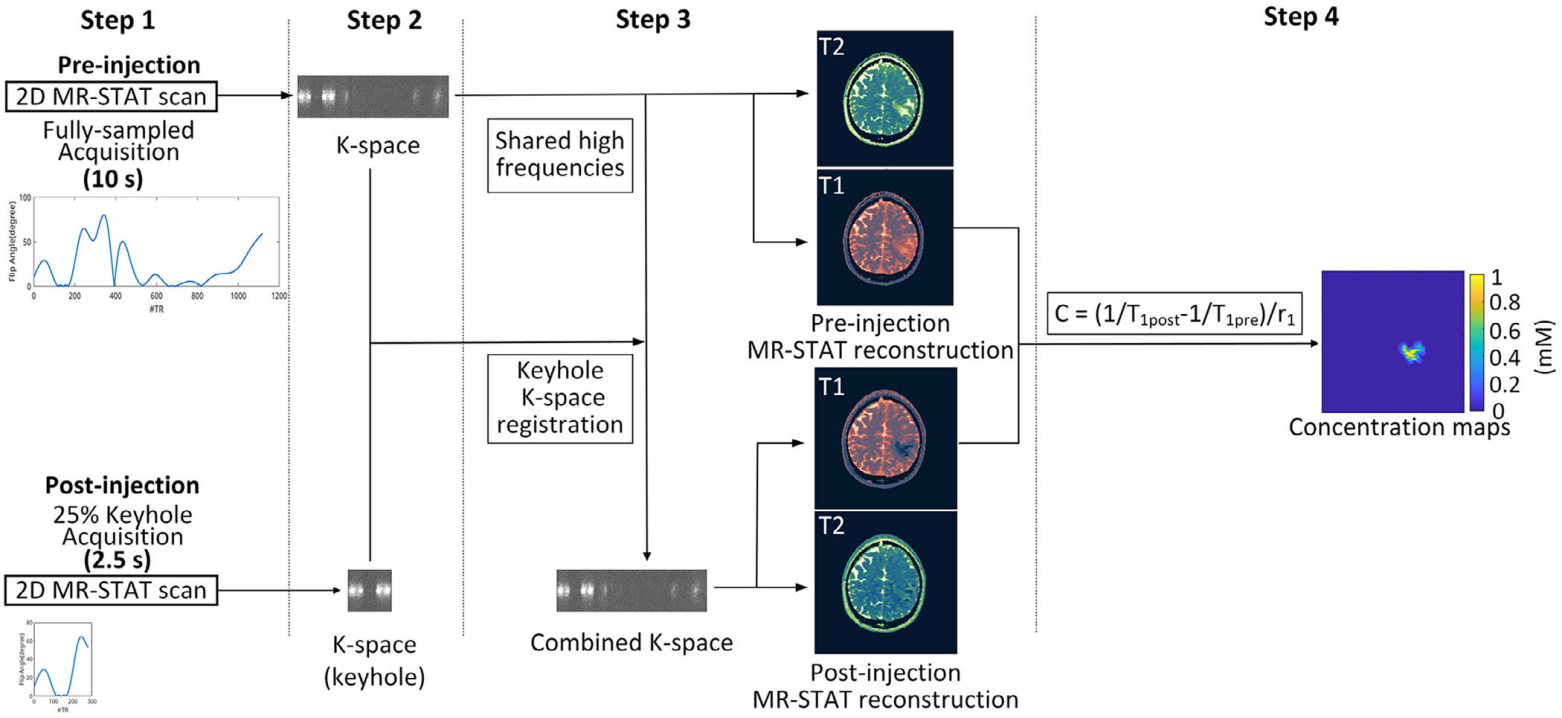
Flowchart of the proposed MR‐STAT protocol. MR‐STAT, Magnetic Resonance Spin TomogrAphy in Time‐domain.

The proposed accelerated keyhole‐based MR‐STAT protocol encompasses four key steps (Figure 2):

Step 1: Fully sampled “pre‐injection” and accelerated 25% keyhole “post‐injection” MR‐STAT acquisitions.^11^, ^30^
Step 2: Combination of “pre‐ and post‐injection” k‐space data by integrating the high spatial frequency data from the “pre‐injection” acquisition with the registered keyhole k‐space data of the “post‐injection” acquisition.
Step 3: High‐resolution MR‐STAT reconstruction of *T*_1_ and *T*_2_ maps for both “pre‐ and post‐injection” acquisitions.^30^
Step 4: Reconstruction of GBCA concentration map according to the relaxivity model^31^:
(1) $$C=\left(\frac{1}{T_{1,post}}-\frac{1}{T_{1,pre}}\right)/r_{1},$$

where *C* is the concentration of GBCA, $T_{1,post}$ is the longitudinal relaxation time of the tissue after injecting GBCA, $T_{1,pre}$ is the longitudinal relaxation time before injecting GBCA, $r_{1}$ is the relaxivity of GBCA.

We set the relaxivity $r_{1}$ = 3.6 (L mmol^−1^s^−1^) for gadobutrol based on previous research.^32^

In step 2, low‐resolution images were first generated from the keyhole k‐space data of both pre‐ and post‐injection acquisitions using the fast Fourier transform (FFT). To correct for possible misalignments due to inter‐scan motion, co‐registration of the two datasets was performed in the image domain using MATLAB's inbuilt “imregister” function. The resulting transformation matrix was subsequently applied to the keyhole k‐space data of the post‐injection acquisition to align it with the pre‐injection data.

### 2.3 Phantoms preparation

We first prepared a phantom solution consisting of manganese chloride (Mn^2+^) dissolved in distilled water at 0.1 mM concentration to make *T*_1_/*T*_2_ values close to white/gray matter values.^33^ This substance was used as a “baseline liquid” (*T*_1 _= 978 ms; *T*_2 _= 86 ms). Subsequently, we prepared ten test phantoms comprising five tubes containing the same ‘baseline liquid’ (tube A_1_, B_1,_ C_1,_ D_1,_ E_1_) to mimic pre‐injection tissue and five tubes with different concentrations of gadobutrol (1.0 mM Gadovist, Schering AG, Berlin, Germany) dissolved in the “baseline liquid” to mimic post‐injection changes (tubes A_2_, B_2,_ C_2,_ D_2,_ E_2_).

Clinically relevant GBCA concentration was considered to range between 0.9 and 1.3 mM.^34^ The post‐injection tubes were thus prepared with solution in the range 0.0 to 0.9 mM, specifically: 0.05, 0.09, 0.19, 0.46, and 0.9 mM, for tube A_2_, B_2,_ C_2,_ D_2,_ E_2_, respectively. These actual GBCA concentrations were calculated from the reference *T*_1_ values of each tube, which were obtained by using a spin‐echo inversion‐recovery sequence to exclude experimental imperfections during the preparation of the phantoms.

### 2.4 Experiment design

#### 2.4.1 Experiment 1: Sequence validation

In order to show the performance of the proposed optimized low‐high MR‐STAT protocol, we conducted tests on phantoms by using standard MR‐STAT and the optimized low‐high MR‐STAT sequence (as shown in Figure 1c,d). Each sequence was run on the five phantom tubes *A*_2_–*E*_2_ (see previous section). For this initial test, only the full (pre‐injection) sequence was considered. The mean values and standard deviations (SDs) of reconstructed *T*_1_ and *T*_2_ values were calculated for each tube.

The gold standard values of *T*_1_ were obtained using a single‐echo spin‐echo inversion recovery experiment with TR of 10000 ms, TE of 20 ms, and TIs of [50, 150, 450, 850, 1350, 1850, 2450, 2950, 3450, 3950, 5000] ms. The gold standard values of *T*_2_ were obtained using a single‐echo spin‐echo experiment with TR of 10000 ms, and TEs of [10, 50,80,120,750,240,290,350,450,500,750] ms.

#### 2.4.2 Experiment 2: Synthetic patient data

To retrospectively validate our proposed method on realistic data before implementing it in clinical practice, MR‐STAT tumor datasets^13^ with manually segmented lesions were selected. The quantitative *T*_1_ and *T*_2_ maps from this dataset served as the "pre‐injection" data. Subsequently, we simulated the effect of a GBCA injection by calculating the expected decrease in *T*_1_ and *T*_2_ values in the lesion for five gadobutrol concentration levels (ranging from 0.05 mM to 1.5 mM) according to the following equations^31^:

$$\frac{1}{T_{1,post}}=\frac{1}{T_{1,pre}}+r_{1}\cdot C$$

$$\frac{1}{T_{2,post}}=\frac{1}{T_{2,pre}}+r_{2}\cdot C$$

The relaxivity $r_{2}$ was set to 6.3 (L mmol^−1^s^−1^) for gadobutrol based on previous research.^32^

To enhance the realism of post‐injection simulations on patient data, two specific pre‐injection patient datasets were selected to incorporate typical contrast‐enhancement patterns observed in clinical MRI.^35^ Specifically, meningioma patient data were selected to represent a homogeneous enhancement pattern,^36^ while astrocytoma patient data were chosen to depict ring enhancement pattern,^37^ which is often found in these tumors and suggests central necrosis, a key feature for radiological differentiation of low‐grade versus high‐grade tumors.^38^ Additionally, rigid motion was simulated into these synthetic post‐injection datasets to more closely reproduce clinical scan scenarios.

In order to prevent unrealistic discontinuities, the edges of the tumor region were smoothed by applying a Gaussian filter. These “pre‐injection” and simulated “post‐injection” relaxometry maps were used to numerically simulate and validate the envisioned protocol on a realistic anatomical dataset.

In summary, the synthetic patient data in Experiment 2 included both contrast enhancement, simulated by adjusting *T*_1_ and *T*_2_ relaxation times according to gadobutrol concentration levels, and realistic motion artifacts introduced to mimic potential clinical conditions.

#### 2.4.3 Experiment 3: Prospective in vitro determination of GBCA concentrations

The five baseline tubes (*A*_1_–*E*_1_) were put in a rigid foam holder to mimic pre‐injection data. After the pre‐injection scan, the tubes were removed and replaced in the same position in the holder by the tubes with different concentrations of GBCA (i.e., tubes *A*_2_–E_2_) to emulate the effect of GBCA injection. Post‐injection data were acquired with the accelerated (keyhole) MR‐STAT scan.

Quantitative *T*_1_ and *T*_2_ values for the pre‐ and post–injection acquisitions were first reconstructed. Subsequently, quantitative measurements of GBCA concentrations were conducted by applying Equation (1). For performance validation, we conducted a linear regression analysis between the estimated GBCA concentrations and the reference values.

#### 2.4.4 Experiment 4: Hybrid study

A prepared phantom tube was placed in close proximity of the head of a healthy volunteer to emulate “pre‐injection patient data” (tube A_1_ mimics the lesion before GBCA injection). After the “pre‐injection” scan, the “lesion” tube was replaced with other tubes (tube A_2_, B_2_, C_2_, D_2_, E_2_) with GBCA concentration from 0.05 to 0.9 mM to mimic a “post‐injection” acquisition, reflecting the dynamic range of contrast agent uptake in pathological tissues.

## 3 RESULTS

### 3.1 Experiment 1: Sequence design and optimization

**FIGURE 3:**
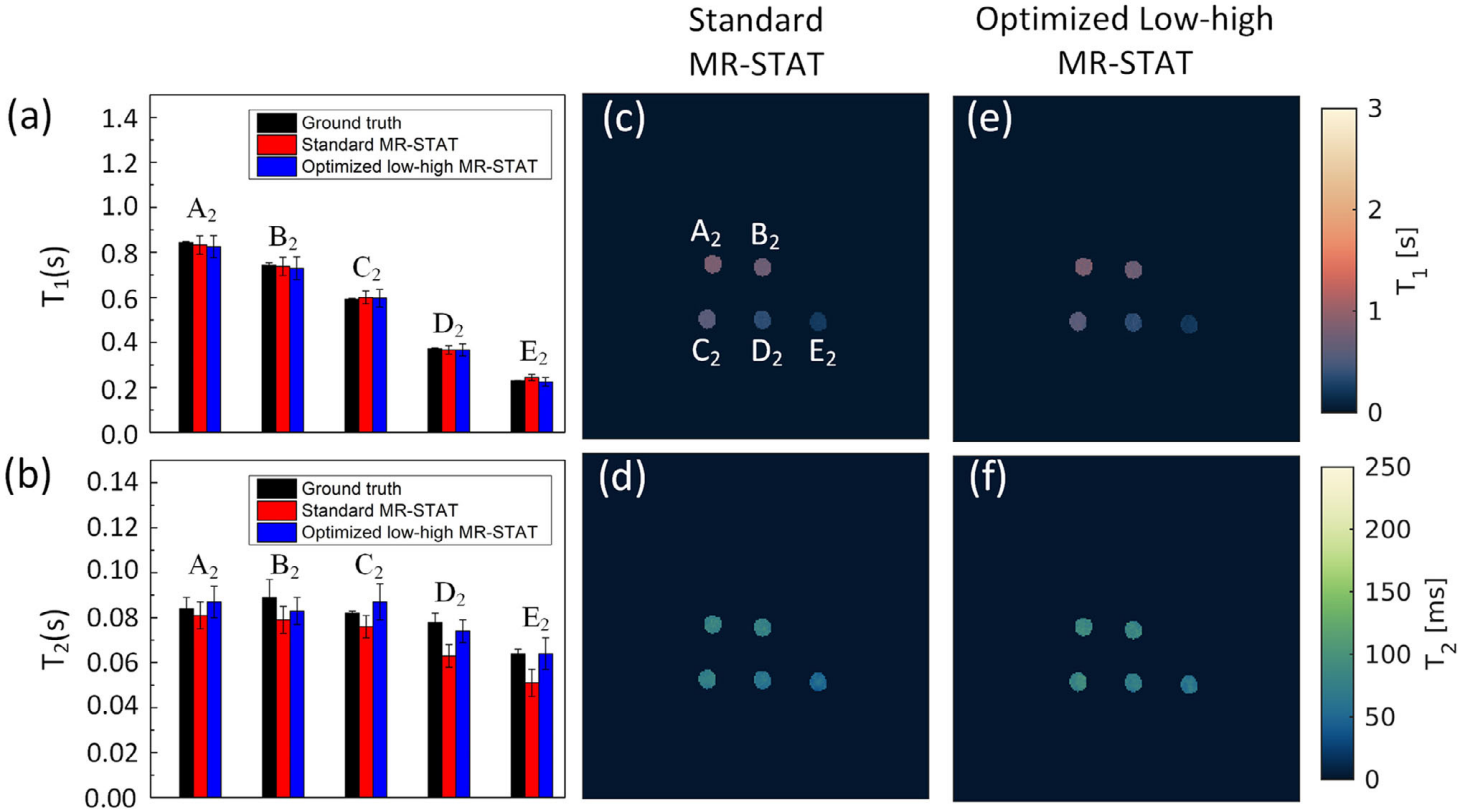
Comparison of standard MR‐STAT and optimized low‐high MR‐STAT measurements. (a, b), the *T*_1_ and *T*_2_ values from gold standard measurements, standard MR‐STAT, and optimized low‐high MR‐STAT. (c) and (e), *T*_1_ maps acquired from standard MR‐STAT, and optimized low‐high MR‐STAT; (d) and (f), *T*_2_ maps acquired from the corresponding measurements. Error bars represent the standard deviation of the measurements. MR‐STAT, Magnetic Resonance Spin TomogrAphy in Time‐domain.

Figure 3 presents the *T*_1_ and *T*_2_ values corresponding to the five tubes obtained from the two fully sampled sequences. In addition, we provide gold standard values which were acquired with a spin‐echo inversion‐recovery sequence. It shows that the optimized low‐high MR‐STAT method has the ability to accurately reconstruct *T*_1_ and *T*_2_ values comparable to gold standard measurements, while maintaining a similar noise level (standard deviation) as the standard MR‐STAT protocol.

### 3.2 Experiment 2: Synthetic patient data validation

**FIGURE 4:**
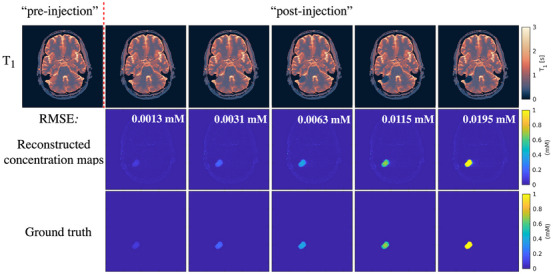
*T*_1_ and GBCA concentration maps of synthetic meningioma patient data with different simulated GBCA concentrations by applying the proposed protocol. The values in the reconstructed concentration maps are the RMSE values between the reconstructed results and the ground truth. GBCA, gadolinium‐based contrast agent; RMSE, root mean square error.

**FIGURE 5:**
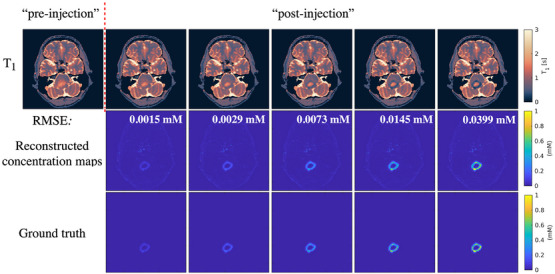
*T*_1_ and GBCA concentration maps of synthetic astrocytoma patient data with different simulated GBCA concentrations by applying the proposed protocol. The values in the reconstructed concentration maps are the RMSE values between the reconstructed results and ground truth. GBCA, gadolinium‐based contrast agent; RMSE, root mean square error.

Figures 4 and 5 display the *T*_1_ maps and concentration maps for “pre‐injection”, “post‐injection” with different concentrations from the representative slice of the synthetic meningioma patient data and synthetic astrocytoma patient data, respectively. To demonstrate the consistency of the proposed multi‐parametric protocol before and after gadolinium injection, the *T*_2_ maps are shown in Figures S1 and S2 alongside the *T*_1_ maps. The root mean square error (RMSE) values between the reconstructed results and ground truth indicate the effectiveness of the proposed method in accurately detecting concentration changes.^39^ Additionally, for one case of the synthetic dataset, we also presented the reconstructed results without registration to highlight the impact of motion correction (as shown in Figure S3).

### 3.3 Experiment 3: Prospective in vitro determination of GBCA concentration

**FIGURE 6:**
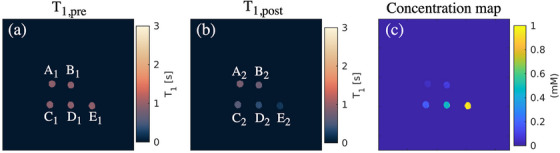
GBCA concentration map of phantom tubes containing contrast agent solutions. (a)–(b), pre‐ and post‐injection *T*_1_ maps acquired, respectively, with fully sampled and under‐sampled (keyhole factor of 25%) MR‐STAT sequences; (c), the concentration maps calculated from *T*_1pre_ and *T*_1post_. GBCA, gadolinium‐based contrast agent; MR‐STAT, Magnetic Resonance Spin TomogrAphy in Time‐domain.

In vitro validation of the optimized low‐high MR‐STAT measurements for estimating GBCA concentrations is shown in Figure 6. The corresponding *T*_2_ maps are shown in Figure S4.

**FIGURE 7:**
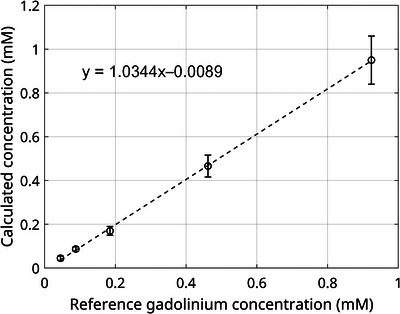
Correlation plot comparing the estimated GBCA concentration to reference values. Error bars represent standard deviations in the corresponding tubes. GBCA, gadolinium‐based contrast agent.

The measured GBCA concentrations are very close to the reference values, as evidenced by the linear regression analysis yielding a slope of 1.03 and an intercept of 0.009 (Figure 7).

### 3.4 Experiment 4: Hybrid study

**FIGURE 8:**
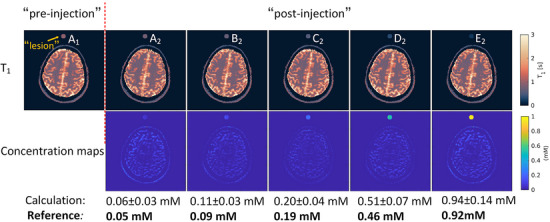
*T*_1_ and GBCA concentration maps of a healthy volunteer with different phantom tubes (surrogate for lesion) by applying the proposed protocol. The values below the figure are the estimated GBCA concentration and the corresponding reference values of the phantom tubes. GBCA, gadolinium‐based contrast agent.

In Figure 8, the *T*_1_ and GBCA concentration maps obtained from the hybrid in vivo/in vitro study provide a comprehensive visualization of tissue properties and GBCA distribution. The corresponding *T*_2_ maps are shown in Figure S5. Upon comparing the estimated GBCA concentration with the reference values, it demonstrates the proposed method's accurate detection of concentration changes.

## 4 DISCUSSION

In this study, we demonstrated that GBCA concentrations in the range of 0.05–0.9 mM could be measured with an optimized and accelerated MR‐STAT protocol. The proposed method incorporated a low‐high sampling pattern for pre‐ and post‐injection acquisition, where the center k‐space data for these two acquisitions share the same optimized RF pulse sequence and the same encoding pattern. This design aims to make the two acquisitions as consistent as possible, thereby ensuring that changes in *T*_1_ map primarily reflect the concentration of GBCA and are not influenced by other confounding factors.^25^, ^26^, ^27^, ^28^ The quantitative *T*_1_ and *T*_2_ maps from the newly proposed low‐high optimized protocol are comparable with those from the standard MR‐STAT.^11^ Concentration estimation demonstrated high accuracy in the proposed method when compared to the reference values.

While conventional assessment of contrast enhancement relies on subjective, qualitative interpretation, this method could enable the acquisition of an objective, quantitative index to delineate the contrast enhancement characteristics of the lesion, thereby facilitating and/or accelerating the radiological inspection. Notably, as conventional concentration estimation from *T*_1_‐weighted imaging can be problematic due to the *T*_2_ shortening effect at high GBCA concentrations, our estimation method based on *T*_1_ mapping and the relaxivity model (Equation 1) circumvents this issue. Furthermore, the proposed fast keyhole strategy for post‐injection acquisitions could elucidate the time‐dependent concentration changes in lesions and thus provide crucial information in contrast‐enhanced scans with a temporal resolution of 2.5 s, comparable to that in DCE clinical scans.^40^, ^41^, ^42^, ^43^

This study has some limitations. While we retrospectively attempted to enhance the realism of post‐injection simulations on patient data by incorporating contrast‐enhancement patterns observed in clinical MRI and prospectively validated the method at the proof‐of‐concept level through a hybrid human/phantom study, a clinical study will still be necessary to thoroughly validate the proposed method in vivo. When dealing with in vivo data, bulk patient motion during and between scans may lead to misregistration of images before and after GBCA administration. Without using the properly registered *T*_1pre_ and *T*_1post_ maps, the calculated apparent GBCA concentration could be erroneously estimated. Registration based on 2D datasets may not be adequately correct for tilting, out‐of‐slice movement, resulting in inferior registration accuracy compared to 3D datasets. In future work, application of accelerated 3D acquisitions to the brain could refine registration between pre‐ and post‐injection images, facilitating the development of a more robust method for estimating GBCA concentrations.^44^ The proposed keyhole‐like workflow aligns well with 3D MR‐STAT due to its Cartesian encoding, though it may pose challenges for other fast multiparametric methods like MRF, which typically use spiral or radial encoding schemes. Another potential advantage of 3D acquisitions is the prospect of better performance in terms of scan time reduction. For 2D acceleration, we employed an acceleration factor of 4, taking into account the necessity for the post‐injection acquisition to retain sufficient encoding information, that is, time for the MR‐signal to evolve, to accurately estimate *T*_1_ and *T*_2_ values, and to capture meaningful quantitative changes. In contrast, 3D acquisitions offer greater flexibility due to the presence of two phase‐encoding directions. This is achievable through the application of the keyhole undersampling strategy in the additional phase‐encoding direction. Concretely, applying keyhole factor of 4 in both phase encoding directions, we achieve accelerations of 16, reducing the acquisition time to approximately 20 s, as estimated from the fully sampled reference acquisition.^44^ Other methods available in clinical practice, such as compressed sensing,^45^ can achieve smaller acceleration factor, but they can probably be further accelerated when combined with our approach.

## 5 CONCLUSION

The accurate quantification of GBCA concentration can be achieved with high accuracy in a clinically feasible time using an optimized and accelerated MR‐STAT protocol. Further clinical studies are necessary to validate the method on patients.

## CONFLICT OF INTEREST STATEMENT

The authors declare no conflicts of interest.

## Supporting information



Supporting Information

## Data Availability

The data that support the findings of this study are available upon reasonable request from the authors.
