# Supplementary material for: Efficient estimation of gadolinium‐based contrast agent concentration using transient‐state keyhole MR‐STAT
Source: Med Phys. 2025 Nov 19;52(12):e70155. doi: 10.1002/mp.70155 (PMC12630068; doi:10.1002/mp.70155)
Supplement: Supplementary file 1 — Supporting Information [file MP-52-0-s001.pdf]

Supplementary material to

Efficient Estimation of Gadolinium-based Contrast Agent Concentration Using Transient-state Keyhole MR-STAT

Fei Xu[1], Edwin Versteeg[1], Hongyan Liu[1], Miha Fuderer[1], Oscar van den Heide[1], Wybe J. M. van der Kemp[2], Cornelis A.T. van den Berg[1], and Alessandro Sbrizzi[1]

[1]Computational Imaging Group for MR diagnostics & therapy, Center for Image Sciences, UMC Utrecht, Utrecht, Netherlands

[2]Department of Radiology, University Medical Center Utrecht, Utrecht, The Netherlands

In this section, to demonstrate the consistency of the proposed multi-parametric protocol before and after gadolinium injection, we also include the T_2 maps in **Figures S1–S2** and **S4–S5** alongside the T_1 maps. In addition, we present the reconstructed results without registration for one synthetic dataset case to highlight the impact of motion correction (**Figure S-3**).

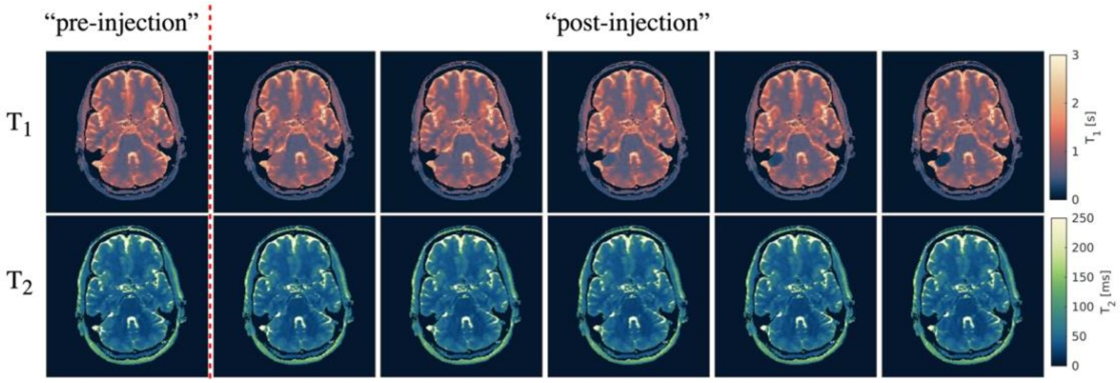

Figure S-1 T_1 and T_2 maps of synthetic meningioma patient data with different simulated GBCA concentrations by applying the proposed protocol.

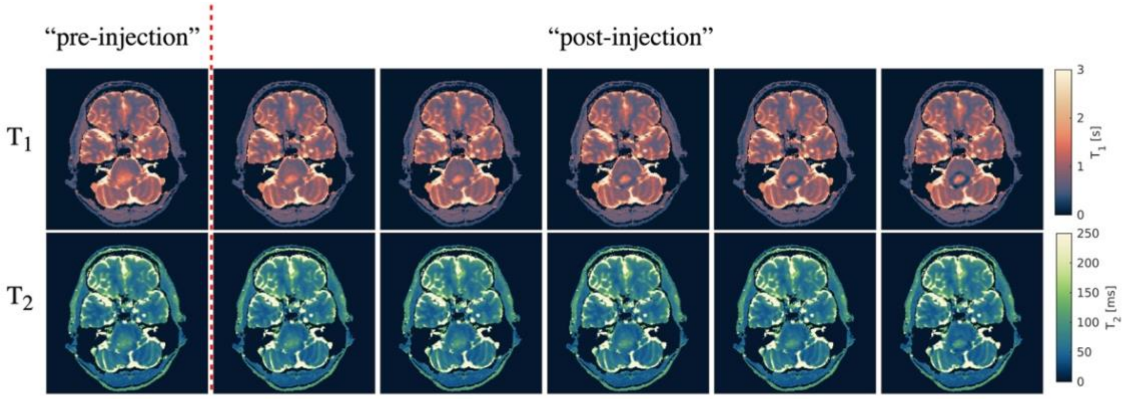

Figure S-2 T_1 and T_2 maps of synthetic astrocytoma patient data with different simulated GBCA concentrations by applying the proposed protocol.

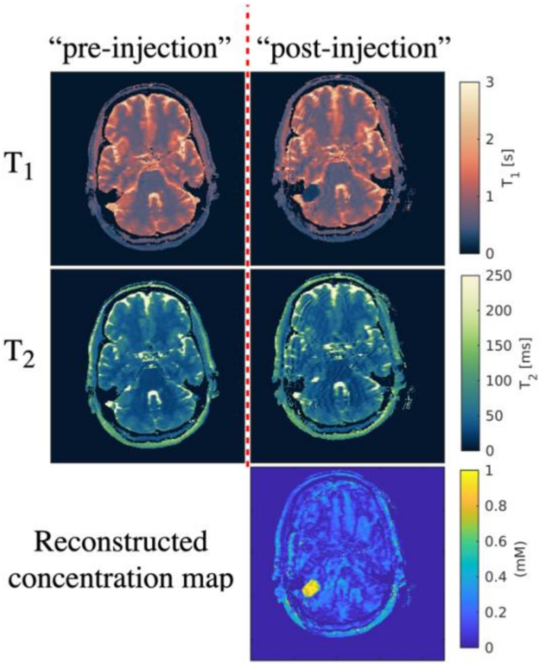

Figure S-3 T_1, T_2 and GBCA concentration maps of synthetic meningioma patient data with a simulated GBCA concentrations by applying the proposed protocol without registration. Compare with Fig. 4 where motion is resolved by inter-scan registration.

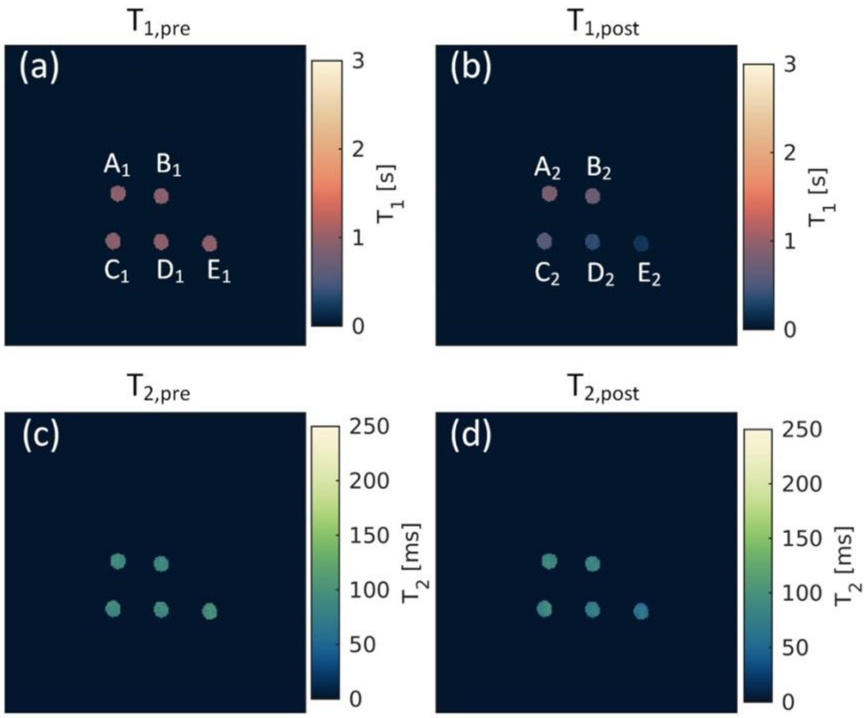

Figure S-4 (a)-(b), pre- and post-injection T_1 maps acquired, respectively, with fully sampled and under-sampled (keyhole factor of 25%) MR-STAT sequences; (c)-(d), pre- and post-injection T_2 maps reconstructed from the same MR-STAT protocol;

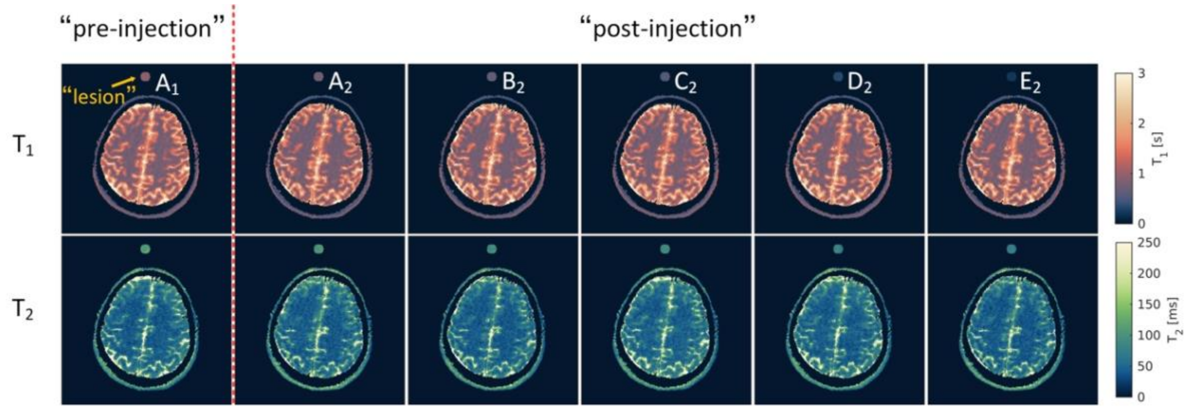

Figure S-5 T_1 and T_2 maps of a healthy volunteer with different phantom-tubes (surrogate for lesion) by applying the proposed protocol.
